# Current trends in rehabilitation of rotator cuff injuries

**DOI:** 10.1051/sicotj/2023011

**Published:** 2023-05-23

**Authors:** Fabio V. Sciarretta, Daniel Moya, Kilian List

**Affiliations:** 1 Clinica Nostra Signora della Mercede 00198 Rome Italy; 2 Department of Orthopedic Surgery, Hospital Británico de Buenos Aires C1280 AEB Argentina; 3 Department of Orthopedic Surgery, Koenig-Ludwig-Haus, University of Wuerzburg Brettreichstraße 11 97074 Wuerzburg Germany

**Keywords:** Rotator cuff, Rehabilitation, Rotator cuff repair, Rehabilitation protocol, Shoulder

## Abstract

Rehabilitation has a fundamental role in the management of rotator cuff pathology whether the final choice is conservative or surgical treatment. Conservative treatment can give excellent results in cases of rotator cuff tendinopathies without rupture, partial tears less than 50% of the thickness of the tendon, chronic full-thickness tears in elderly patients and irreparable tears. It is an option prior to reconstructive surgery in non-pseudo paralytic cases. When surgery is indicated, adequate postoperative rehabilitation is the best complement to obtain a successful result. No consensus has still been established on the optimal postoperative protocol to follow. No differences were found between delayed, early passive and early active protocols after rotator cuff repair. However, early motion improved the range of motion in the short and mid-term, allowing faster recovery. A 5-phase postoperative rehabilitation protocol is described. Rehabilitation is also an option in specific failed surgical procedures. To choose a therapeutic strategy in these cases, it is reasonable to differentiate between Sugaya type 2 or 3 (tendinopathy of the tendon) and type 4 or 5 (discontinuity/retear). The rehabilitation program should always be tailored to the individual patient.

## Introduction

Rehabilitation has a fundamental role in the management of rotator cuff pathology, whether the choice is conservative or surgical treatment. Rehabilitation by itself is enough to solve a significant percentage of habitual pathology. In cases of surgery, it is a very important coadjuvant not only in the postoperative period but also in many cases it is used preoperatively.

Much progress has been made in the conservative therapeutic approach to shoulder injuries, however, for many years the issue has been addressed mostly by surgeons dedicated to the treatment of the pathology of the shoulder girdle [1–4]. In recent years the pendulum has shifted from the surgeon’s decision to other proposals in which the rehabilitator takes centre stage [5]. We believe that both approaches are wrong and that the ideal is teamwork. Unfortunately, frequently the system conspires against teamwork since in many countries the surgeon refers the patient to rehabilitation without having contact or coordination with the rehabilitators.

**Figure 1:**
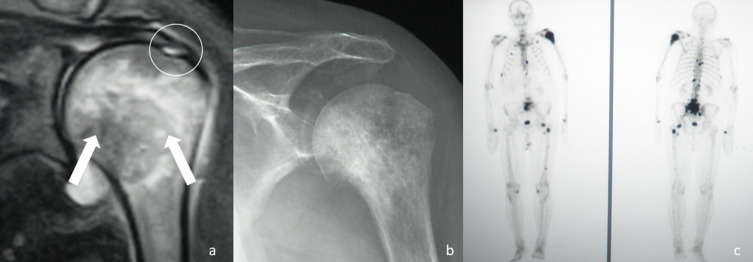
A 67-year-old patient with a history of left shoulder pain for more than one year. He was treated with more than 30 rehabilitation sessions and indicated surgery. (a) Initial image in which the circle marks the rotator cuff lesion. Nobody noticed the alterations of the humeral head. (b) X-rays were requested that had not been requested in the previous consultations in which an alteration in the metaphyseal-epiphyseal region was observed. c) Scintogram showing pathologically increased uptake in different regions of the skeleton and especially in the proximal end of the humerus. The final diagnosis was a metastasis of prostate carcinoma.

It is the surgeon who has at his disposal all the diagnostic tools and manages all the therapeutic options. In many cases, rehabilitation-focused management does not start from a specific diagnosis and simply treats symptoms. This approach, however, is at risk of serious diagnostic errors. The enthusiastic incorporation of ultrasound by physiotherapists and other medical specialities, does not guarantee a correct diagnosis (Figure 1). There may be associated pathology in the humeral head shown in the example that could not be diagnosed sonographically.

Decision-making must be based on the never-so-undervalued “clinical-surgical criteria”. We will never agree that there is “too much medicine in musculoskeletal practice” [5] when there is even the slightest possibility of a severe diagnostic error.

**Figure 2:**
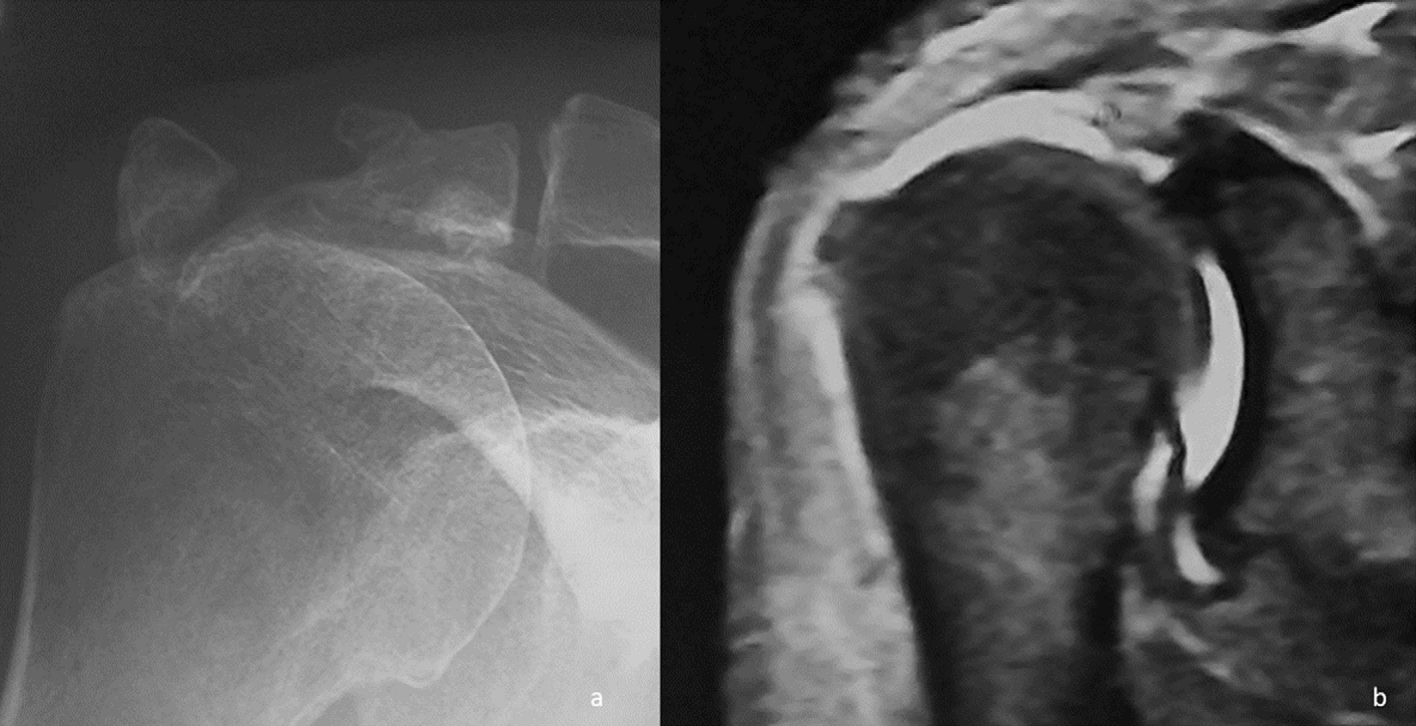
37-year-old patient with a history of right shoulder trauma of 2 months of evolution. He was treated with three local injections of platelet-rich plasma. a) X-rays showed a displaced acromion fracture and magnetic resonance showed a massive rotator cuff tear. Clearly, the initial indication was surgical.

Another factor that has complicated the panorama is the entry into the therapeutic circuit of professionals who, without having a specific training base in musculoskeletal pathology, begin to treat shoulder injuries just because they implement some of the new “regenerative” treatments in vogue (shock waves, platelet-rich plasma, stem cells, hyaluronic acid, etc.). These are situations in which treatment adapts to the professionals’ preferences rather than the patients’ pathology (Figure 2). For the worse, in many of these indications there is no high level of evidence scientific support [6–8]. It ends up putting the value of “trends” or information on social media, over evidence-based medicine. The practice reveals more and more frequently disastrous therapeutic decisions in the name of “regenerative medicine”.

This review aims to analyze the rehabilitation alternatives in three different scenarios: rehabilitation as the unique treatment for rotator cuff tendinopathies, postoperative rehabilitation as a complement to tendon repair surgery, and the role of rehabilitation in failed rotator cuff surgeries, using a consensus approach between rehabilitators and surgeons.

## Rehabilitation as an alternative to surgical treatment

The initial indication for conservative treatment in rotator cuff pathology includes tendinopathies without rupture, partial lesions less than 50% of the thickness of the tendon, chronic full-thickness tears in elderly patients, and irreparable tears as an option prior to reconstructive surgery in non-pseudo paralytic cases.

It has been demonstrated that in the case of tendinopathies without rupture, the results of surgery and conservative treatment are similar [9, 10]. Even having described a subacromial decompression surgical technique, Rockwood [3] always emphasized the need to exhaust the conservative option before proceeding with surgery. He was one of the pioneers in the use of elastic bands more than 30 years ago and highlighted the value of supervised home-based programs [3, 4]. This was supported by numerous studies in recent times [11–13].

The Rockwood program is based on four fundamental goals: 1) pain control, 2) mobility recovery, 3) the development of muscular strength and 4) long-term maintenance.

The strengthening process involves the scapular stabilizers, the humeral head depressors and finally the deltoid muscle. These postulates today sound too basic in relation to the degree of rehabilitation development reached and the appearance of new, more sophisticated approaches, however in daily practice they are not always applied.

In the case of partial rotator cuff injuries that do not exceed 50% of the thickness of the tendon, it is justified to exhaust the chances of a conservative treatment since the risk of fatty infiltration, muscular atrophy, and severe tear extension are relatively low [14]. Conservative treatment of partial rotator cuff injuries is similar to that for patients with tendinopathies with no tears [15].

In a total series of 76 patients with partial rotator cuff injuries, Lo et al. [16] treated conservatively 37 cases with significant clinical improvement. At a mean of approximately 4 years follow-up, 91% of these cases were still satisfied with the nonoperative treatment result [16]. Predictors of poor results with conservative treatment have been described, including bursal location, dominant side, and involvement of more than 50% of the tendon thickness [17].

The initial indication in patients older than 70 years old with full-thickness rupture and low functional demands is conservative, not only because of the high rate of positive results but also because the biological healing capacity of the repaired tendon is greatly diminished. The indication for surgery in the elderly is linked to pain control when conservative treatment has failed. Some authors have proposed extending the conservative indication to younger patients with specific tears. Kukkonen et al. in a randomized controlled trial [18] found no difference comparing operative treatment with conservative treatment results regarding small, nontraumatic, single-tendon supraspinatus tears in patients older than 55 years. However, in a more recent study, Moosmayer et al. [19] concluded that at a 10-year follow-up, tendon repair is superior to physiotherapy in the treatment of small and medium-sized rotator cuff tears (RCT).

In cases of full-thickness ruptures in young and active patients, and especially when they are acute, the results of surgery are superior to conservative treatment [12, 20].

In irreparable massive ruptures, rehabilitation also has an important role, especially in cases that do not present pseudoparalysis and that have controllable painful symptoms [21–23].

Christensen et al. [21] reported that patients with irreparable RCT following a five-month exercise protocol gained greater function in their symptomatic shoulder, with less pain, and better quality of life.

Levy et al. [22] used a structured deltoid rehabilitation program in medically unfit elderly patients with massive RCT. At a minimum 9 months follow-up 14 of 17 patients had a significant improvement in the Constant score.

Patients with a nonoperatively managed, moderately symptomatic massive RCT can maintain satisfactory shoulder function for at least four years [23].

## Postoperative rehabilitation of rotator cuff repair

An RCT can be surgically treated with an open, mini-open or arthroscopic repair. According to Liu et al. [24], arthroscopic rotator cuff repair (ARCR) allows faster recovery and good cosmetic results and is frequently performed in conjunction with ancillary procedures such as long-head biceps tenotomy-tenodesis, subacromial decompression, distal clavicle resection.

To provide a secure and customized rehabilitation treatment, it is critical to understand the different timings and biological events of the healing sequence [25]. Following surgery, tissue repair proceeds through three overlapping phases: inflammation (0–7 days), proliferation (5–25 days) and maturation-remodelling (>21 days to 6 months) [26, 27]. Collagen type III, is present in a high percentage of scar tissue, possibly accounting for weaker tendon healing and reduced tendon strength [28].

The main objectives of the rehabilitation process after ARCR are promoting healing of the repaired tendons, restoring function and regaining the range of motion and strength of the shoulder while minimizing shoulder stiffness and muscle atrophy.

To date, the literature presents just a few high-level evidence-based studies focusing in detail on postoperative rehabilitation regimens and no consensus has still been established on the optimal postoperative protocol to follow [29].

Three postoperative protocols have been described after ARCR: strict immobilization, early passive motion and early assisted active motion. It might be reasonable to assume that early motion, whether passive or active, increases the range of motion (ROM) after ARCR, while the risk of re-tear could be significantly higher if compared to delayed motion. On the other hand, immobilization could decrease the likelihood of re-tear, but could also result in shoulder stiffness, which can cause functional limitations, pain, and frustration.

For convenience, results will be subdivided into early versus delayed motion analyzing a range of motion, functional outcome, repair integrity, tear size, loading and strengthening (Table 1).

**Table 1:** Comparison between early and delayed mobility protocols results after rotator cuff repair surgery (ROM: range of motion).

	ROM	Functional outcomes	Strength	Repair integrity
Early motion	Better ROM recovery	No significant differences at 6-month, 1- and 2-year follow-ups.	No significant difference with the different protocols.	No significant difference with the different protocols.
Delayed motion	Lower ROM at 6 months and 1 year.			

The early passive motion protocol includes passive shoulder ROM activities starting on the first postoperative day. The delayed passive motion protocol consists of sling immobilization, except pendulum exercises, for the first 4–6 postoperatively weeks.

The current, best available evidence [30–33] suggests that delayed passive motion (DPM), early passive motion (EPM) and early active motion (EAM) were equivalent in terms of long-term ROM recovery, functional outcomes and healing rate.

At 6 postoperative months, strict immobilization was associated with lower flexion, abduction, external and internal rotation than EPM or EAM [28, 29, 32, 33].

Strict immobilization reduced flexion at 1 year compared to passive or active motion [28, 29, 33].

At a 2-year follow-up, no significant differences between the two EPM and EAM protocols in terms of ROMs were found, however, this result may be influenced by the lack of studies with long-term follow-up [28].

A most recent meta-analysis [28–30, 33] revealed no significant differences in the functional outcomes between the two different rehabilitation protocols, at 6-month, 1- and 2-year follow-ups. However, although not statistically significant, early motion rehabilitation protocol was associated with higher scores than delayed motion. Likewise, regarding strength recovery, both groups improved in strength in terms of flexion, abduction, internal and external rotations and no statistical differences between protocols were found for any tests at any follow-up [33].

No statistical difference was found regarding shoulder stiffness at 2 years follow-up either [34].

Anatomic failure, non-healing or re-tear, after ARCR is not uncommon (25–60%) and it tends to occur in the first 3–6 months after surgery [35]. The most significant reviews [28–30, 33] demonstrated that, at a minimum 1-year follow-up, there is no significant difference in the results obtained with the different rehabilitation protocols, even if strict immobilization showed the highest values versus EPM and EAM.

The risks of failure after ARCR are well documented and include in addition to larger tear size [35–38], poor tissue quality [38], age, fatty infiltration and atrophy of rotator cuff muscles, smoking, hypercholesterolemia, arterial hypertension, alcohol consumption and diabetes [35, 39–42]. These factors should be taken into account when choosing the most appropriate rehabilitation protocol.

The literature lacks strong evidence about the correlation between different postoperative rehabilitation protocols and RCT size.

Raschhofer et al. [43], investigated the effect of early low-intensity isometric loading and EPM during the first 2–6 weeks of rehabilitation and concluded that low isometrics load could stimulate scar and tendon remodelling, contributing to improved outcomes.

According to current literature [28, 33] and AAOS (American Academy of Orthopaedic Surgeons) and ASSET (American Society of Shoulder and Elbow Therapists) guidelines [35] we propose our 5 phases rehabilitation protocol (Table 2) for patients undergoing rotator cuff repair adjusted according to tear size and quality of tendons tissue.

- Phase 1 (weeks 0–2): strict immobilization. Distal hand and wrist active motion is allowed.
- Phase 2 (weeks 2–6): continue immobilization. A protected passive range of motion (PROM) is initiated by the patients in the scapular plane. It is allowed the elbow’s active range of motion (AROM) with the arm at the side. During weeks 4–6, it is allowed to move from PROM to an active-assistive range of motion (AAROM) by supervised physiotherapy. Criteria to advance to the next step are pain-free PROM, external rotation >30° and forward flexion >120°.
- Phase 3 (weeks 6–12): progress from AAROM to AROM with <15% supraspinatus electromyographic activity level. Criteria to advance are full and pain-free PROM and AROM without compensation, no shoulder “shrug”, and pain-free isometric exercises.
- Phase 4 (weeks 12–20): strengthening and endurance with 30–49% supraspinatus electromyographic activity level.
- Phase 5 (weeks 20–26): strengthening with >50% supraspinatus electromyographic activity level.

**Table 2:** Five-phase postoperative rehabilitation protocol (AROM: Active range of motion, AAROM: Active-assistive range of motion, PROM: Protected passive range of motion).

	Weeks	Protocol
Phase 1	0–2	Strict immobilization. Distal hand and wrist active motion.
Phase 2	2–6	Continue immobilization. Protected passive range of motion. Elbow’s AROM. Move from PROM to AAROM at 4–6 weeks.
Phase 3	6–12	Progress from AAROM to AROM.
Phase 4	12–20	Strengthening and endurance.
Phase 5	20–26	Strengthening.

### Rehabilitation after failed rotator cuff repair

**Figure 3:**
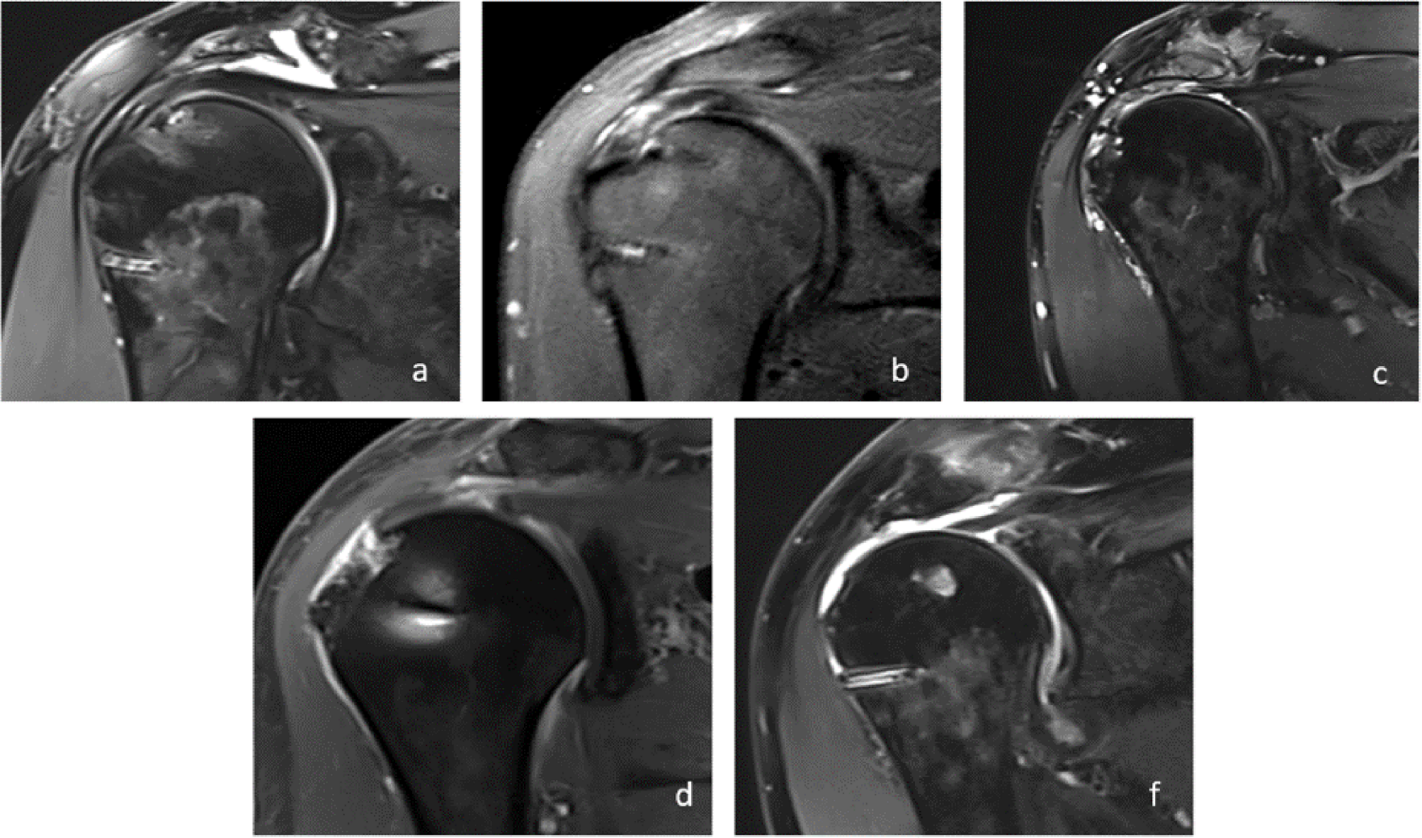
Sugaya’s classification rates (a) type 1 as fully healed, (b) type 2 and (c) type 3 as persistent signs of tendinopathy or (d) type 4 minor and (e) type 5 major as discontinuity [2].

Rotator cuff repair may be considered “failed” for various reasons: persisting pain or loss of function, inability to return to work or previous sports level, anterosuperior escape, cuff tear arthropathy or perioperative complications such as infection. With the exception of infection, most of these conditions are due to retears. Perioperative complications aside, most of these problems are due to retears of the rotator cuff. Retear rates depend on patient-related (age, larger tear size, fatty infiltration) and not patient-related (postoperative rehabilitation protocol, surgical techniques, and procedures) factors. They are reported inconsistently and may be around a considerable 20% [44]. This inconsistency is aggravated by the unclear definition of retear. Sugaya’s classification (Figure 3) is commonly used and seems suited as it predicts motoric integrity and function after rotator cuff repair [45]. However, the inter-observer agreement was only fair [45, 46].

**Figure 4:**
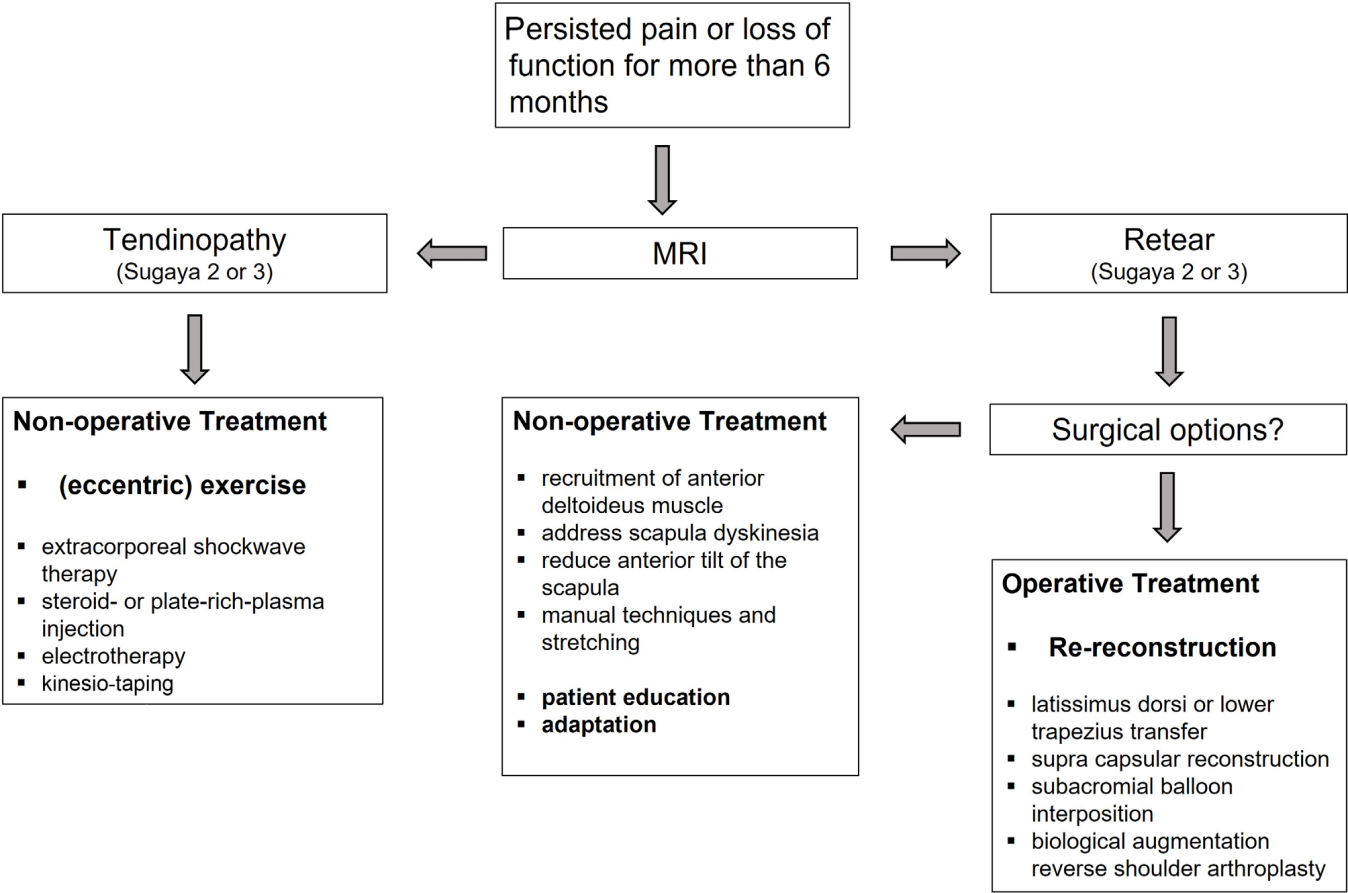
Treatment algorithm for failed rotator cuff tear. With persistent pain or loss of function for more than 6 months an MRI should be performed. Based on the Sugaya classification [2] tendinopathy and retear are differentiated. Non-operative treatment for tendinopathy should include eccentric exercises to promote tendon healing. When a retear is confirmed, surgical options are evaluated. If surgery is not favourable, pillars for non-operative treatment are compensation, adaption, and patient education.

To choose a therapeutic strategy (Figure 4), it is reasonable to differentiate between Sugaya type 2 or 3 (tendinopathy of the tendon) and type 4 or 5 (discontinuity/retear) [45].

Persisting tendinopathy after rotator cuff repair (Sugaya 2 or 3) does not seem to correlate with pre-existing tendinopathy in concordance with the initial [47] but may be promoted by the immobilization after the initial repair [48]. In the case of symptomatic persisting tendinopathy, surgical options are limited. A variety of therapeutic options exists, including (eccentric) exercise [49], extracorporeal shockwave therapy [50], steroid- [51] or plate-rich-plasma (PRP) injection [52], electrotherapy [53] or kinesio-taping [54]. However, the key to tendon regeneration is the mechanical loading of the tendon to initiate proliferation and improvement of collagen structure [48, 55, 56]. While evidence exists for the lower extremities, especially for the achilles and patella tendon [57–61], there is a lack of research on rotator cuff tendinopathy. This is mainly caused by an insufficient methodological design of the available studies. Most studies report on impingement or subacromial shoulder syndrome, but very few focus on tendinopathy of the rotator cuff [49, 62, 63]. Consequently, the evidence for the treatment listed above is either weak or lacking completely. In any case, healing demands time and therefore patient education is most important [64].

In contrast to the first group, in patients with retear (Sugaya 4 or 5), the treating physician or surgeon may consider surgical options such as a further attempt of reconstruction, latissimus dorsi or lower trapezius transfer, supra capsular reconstruction, subacromial balloon interposition, biological augmentation or reverse shoulder arthroplasty. For rotator cuff repairs, it was shown that patients undergoing nonoperative treatment had improved outcomes in the initial follow-up period compared with patients undergoing a surgical procedure, but this trend reversed in the longer term [65, 66]. This might also count for re-repairs making revision still favourable, despite the higher retear rate.

If re-operation is not in favour, non-operative treatment vastly depends on the type of retear. In that perspective, it is not the pain that correlates with the type of re-rupture but the functional impairment [67, 68]. Walch et al. differentiated five tear pattern and investigated the resulting functional impairment [67]. Their study found the majority of ruptures to be orientated anterosuperior [67]. In concordance with the multicenter study cited above, pain did not differ between the groups [67]. But the rupture pattern had a major impact on the ability for shoulder flexion that was worse for the anterosuperior type of rupture, whereas patients with posterosuperior ruptures had the worst external rotation [67]. Walch et al. hypothesized that especially patients with a more posterior tear pattern may well profit from non-operative treatment [67].

In contrast to patients with postoperative tendinopathy, the rehabilitation cannot aim for tendon healing, instead, it should focus to compensate for the missing part of the rotator cuff. Levy et al. propose that the anterior deltoid is the pillar for successful rehabilitation [22]. Following this theoretical principle, the deltoid muscle as a synergist of the rotator cuff can best be trained when the arm is flexed. To improve scapula position relaxation of the pectoralis major muscle, upper trapezius muscle and levator scapulae muscle may address scapula dyskinesia and therefore aid to relieve pain [67, 69]. The anterior tilt of the scapula is reduced by training the serratus anterior and the external rotators are strengthened [67]. Manual techniques and stretching help to recenter the joint and restore arthroceptive conditions [67]. Beside this multimodal therapy, patient education and patients’ adaption of their life with a functionally impaired shoulder is crucial [70].

## Conclusions

Rehabilitation has a fundamental role in the management of rotator cuff pathology. Numerous cuff injuries can be treated with good results by applying for only an adequate rehabilitation program.

The role of rehabilitation in the postoperative period of rotator cuff repairs is essential. There are different protocols with theoretical advantages and disadvantages, but in practice, it has not been possible to demonstrate statistically significant differences between them.

In cases of failed repair surgeries and complications, except in infections, rehabilitation is a primary indication.

In all scenarios, it is very useful to follow a defined rehabilitation protocol adapted to the characteristics of each patient, including a supervised home program.

## Conflict of interest

All authors certify that they have no financial conflict of interest (e.g., consultancies, stock ownership, equity interest, patent/licensing arrangements, etc.) in connection with this article.

## Funding

This research did not receive any specific funding.

## Ethical approval

Ethical approval was not required.

## Informed consent

This article does not contain any studies involving human subjects. Written informed consent was obtained from all patients and/or families of the cases presented in Figures 1, 2 and 3.

## Authors contributions

All authors contributed to writing original drafts, reviewing and editing. Fabio V. Sciarretta wrote “Rehabilitation after failed rotator cuff repair” section. Daniel Moya wrote “Introduction” and “Rehabilitation as an alternative to surgical treatment” sections. Kilian List wrote “Rehabilitation after failed rotator cuff repair” section.
